# Analysis of air pollution in Fenwei Plain in China based on functional spatial autoregressive combined model

**DOI:** 10.1371/journal.pone.0283336

**Published:** 2023-05-12

**Authors:** Jinxian Tang, Xiaoping Shi, Xijian Hu

**Affiliations:** College of Mathematics and System Science, Xinjiang University, Urumqi, Xinjiang, China; Northeastern University (Shenyang China), CHINA

## Abstract

The Fenwei Plain is listed as one of the most serious air pollution regions in China, along with Beijing-Tianjin-Hebei and Yangtze River Delta regions. This paper proposed a functional data analysis method to study the environmental pollution problem in the Fenwei Plain of China. Functional spatial autoregressive combined (FSAC) model with spatial autocorrelation of both the response variable and error term is developed. The model takes the *SO*_2_ concentration of Fenwei Plain as the dependent variable and the dew point temperature as the independent variable and realizes the maximum likelihood estimation using functional principal component analysis to obtain the asymptotic properties of parameter estimation and the confidence interval of the slope function. According to the findings of the empirical analysis of the Fenwei Plain, the *SO*_2_ concentration has significant seasonal characteristics and has decreased year over year for three years in a row. Winter is the season with the highest concentration on the Fenwei Plain, followed by spring and autumn, while summer is the season with the lowest concentration. Winter also has a high spatial autocorrelation. The FSAC model is more effective at fitting the concentration and dew point temperature of the Fenwei Plain in China because its mean square error (MSE) is significantly lower than that of the other models. As a result, this paper can more thoroughly study the pollution problem on the Fenwei Plain and offer guidance for prevention and control.

## 1. Introduction

In recent years, with the rapid economic and industrial development, China’s air pollution emissions have increased significantly. As one of the countries with the highest level of air pollution and the largest population, the disease burden and health economy caused by ambient air pollution in China are considered much higher than those in other regions of the world. Therefore, China’s environmental problems have received extensive attention from scholars, and the study of air pollution is urgent [1, 2]. There is great regional diversity in China, so studying air pollution from a regional perspective is beneficial to draw more accurate conclusions [3]. With the development of urbanization in China, air pollution is influenced not only by independent cities but also by other regional; the phenomenon has been classified as air pollution spillover [4]. Li and Zhou [5] explored the factors influencing urban form and air quality in 288 prefecture-level cities in China. Studies using correlation analysis and regression analysis have found that air pollutants and meteorological parameters such as air temperature, air pressure, humidity, and precipitation are also related [6]. Through reviewing the literature, we found that most of the current studies use classical spatial econometric models to analyze the influencing factors of air pollution and model extensions based on such models. Huang [7] applied the panel Spatial Durbin (SDM) models considering the potential spatial spillover of *SO*_2_ emissions using panel data from more than 30 provinces, although the spatial correlation of the error term is considered based on the original spatial econometric model, the *SO*_2_ concentration is actually a possible spatial pollution spillover, so the omission of the spatial lag term in the model may not be of strong scientific utility. Lou et al. [8] and Jiang et al. [9] introduced different adjacency matrices separately in the spatial econometric model to study the SO2 and the spatial correlation between different industries. To explain the problem of spatial heterogeneity, Li et al. [10] analyzed the interaction of socioeconomic factors and meteorological indicators on pollution impacts using the spatial econometric model, the geographically weighted regression model (GWR), and the generalized additive model (GAM). Current studies on air pollution are analyzed by spatial statistical methods and the GWR model that have higher explanatory power than traditional linear models. Since observations of air pollution data may be spatially correlated, the assumption of independent homogeneous distribution for the model should be avoided in this case, so spatiotemporal statistical models with spatial covariates and time dependence can be further considered. Hassan et al. [11] incorporated an autoregressive integrated moving average (ARIMA) and a Monte Carlo simulation to propose a new Air Pollution Global Risk Assessment (APGRA) prediction model for spatial quality indexes of spatial correlations to address practical issues.

*SO*_2_ pollution is more serious in northern China, and studies have shown that: coal, population, area and thermal power all have a large impact on the *SO*_2_ concentration, with changes in coal consumption having a more significant impact on the national *SO*_2_ concentration [12]. The energy structure of the Fenwei Plain, including Shaanxi, Shanxi, and Henan provinces, is dominated by coal, which is burned in huge quantities, and the consumption of large goods such as coal and coke is dominated by road transport, which leads to the same problem of motor vehicle emission pollution. In addition, the topography of the Fenwei Plain is not conducive to the diffusion of pollutants, resulting in a high rate of particulate matter exceedance in the Fenwei Plain [13]. Li et al. [14] also found that the topography of the Fenwei Plain is unique in that its regional annual wind speed is low, mountains block it, and sinking airflow on leeward slopes. The above reasons make the Fenwei Plain susceptible to the formation of airflow stagnation zones, which seriously affects the diffusion of pollutants, and the particulate matter pollution, mainly PM2.5 and PM2.5, increases year by year. Therefore, the Fenwei Plain is the region with the highest *SO*_2_ concentration in China and also the region with the higher emission intensity of air pollution sources per unit area, which is a major region for air pollution prevention and control identified by the state, with prominent regional structural pollution problems and heavy industrial structure. Shanxi, for example, has the lowest air pollution efficiency and air pollution control process progress values, and far fewer exhaust gas treatment facilities than other provinces and cities, requiring to implement stricter control and management measures to achieve more remarkable environmental improvement [15, 16]. In China, in response to the year-by-year severe environmental pollution in the Fenwei Plain, the State Council publicly released "The Three-Year Action Plan to Fight Air Pollution" in 2018, and in 2019 the Ministry of Ecology and Environment, together with relevant departments, jointly issued the "Action Plan for Comprehensive Control of Air Pollution in Autumn and Winter of 2019–2020 in Fenwei Plain" in 2019. Although many initiatives have led to a steady and significant decrease in China’s *SO*_2_ concentrations in recent years, there is still room for improvement in Shanxi Province, where annual average concentrations are much higher than in other provinces and cities. During heavy pollution, the probability of particles crossing the boundary layer in Fenwei Plain and its surrounding cities is low, and there are significant differences in particle propagation paths for different seasons and cities, so different control measures need to be taken for different situations according to local conditions [17]. Air pollution in China has a U-shaped distribution in terms of time, with major pollutants varying with the seasons; spatially, it has spatial clustering characteristics, with higher levels in northern China and Xinjiang, which are more vulnerable to being influenced by natural factors [18]. Scholars [19, 20] analyzed the temporal variation and seasonal spatial distribution characteristics of *SO*_2_ concentrations in the Fenwei Plain using column concentration data monitored by the Ozone Monitoring Instrument (OMI) and found that Fenwei Plain has higher levels of *SO*_2_ and *NO*_2_ in autumn and winter and in the flatter topography of the river valley plains. While studying the spatial and temporal distribution of air pollution in the Fenwei Plain, Hao et al. [21] proposed the characteristics of the correlation between pollution concentrations and air temperature, rainfall, and air pressure. The main pollutants of air pollution have the highest correlation with the mean temperature and mixed layer height on the annual scale [22]. So in the subsequent study, we focus on the link between pollutants *SO*_2_ and dew point temperature.

In this paper, nearly 700 monthly hourly dew point temperature data are collected for each city in the Fenwei Plain, so a total of over 90,000 pieces of data information are collected each year for this region of the Fenwei Plain. We introduce Functional Data Analysis (FDA) to handle such complex data in this paper. Functional data analysis was introduced by Canadian statisticians J.O. Ramsay and C.J. Dalzell [23], who usually refer to the data with infinite dimensionality and continuity as functional data, which is widely used in the fields of biology, finance, meteorology and medicine. Ramsay and Sliverman [24] generalized the application of functional data analysis by combining other statistically relevant problems such as principal component analysis, typical correlation analysis, and linear regression with functional data analysis. With the development and promotion of spatial econometrics, Ahmed [25] first proposed the functional spatial autoregressive model and used the maximum likelihood function to obtain the estimation and asymptotic properties of the parameters. Pineda-Rios et al. [26] proposed a functional autoregressive model with autocorrelation of the error term—the simultaneously autoregressive model (FSAR) and obtained the confidence bands of the slope function through the maximum likelihood estimation and asymptotic normality. Aw and Cabral [27] extended the scalar covariates of spatial autoregression to functional covariates and proposed the functional spatial autoregressive combined model (FSAC). Martínez et al. [28] proposed a methodology based on the concept of functional depth to test the presence of outliers in spatial contaminant samples from a functional perspective, which can more powerfully reduce the probability of detecting measurement errors as outliers compared to classical methodologies. Hu et al. [29] developed a functional spatial autoregressive model between the temperature profile and monthly mean *SO*_2_ concentrations and demonstrated that a better fit was achieved using a functional spatial autoregressive model compared to a functional linear model. Besides, no other scholars have used functional regression models to analyze the relationship between air quality data and meteorological data in this region, but the exploration of functional models does not stop here, we propose in this paper to use a new model–functional spatial autoregressive combined model (FSAC) to link with the Fenwei Plain.

This paper combines partial spatial autoregressive models and functional linear models based on functional data and spatial variables and establishes the FSAC model with spatial autocorrelation for both real-valued response variables and error terms to explore air quality in the region not only to make full use of the spatial autocorrelation and high-dimensional continuity of data but also to provide a new way to analyze air pollution in the Fenwei Plain and increase the accuracy of the results. In addition, this paper improves the theoretical part of the model by adding the proof of asymptotic properties, which provides a theoretical basis for the feasibility of the model. Then we verify the spatial correlation of *SO*_2_ concentration in the Fenwei Plain and use the FSAC model to fit the *SO*_2_ concentration and dew point temperature data to analyze the correlation quantitatively between air quality and temperature in the Fenwei Plain. Finally, multiple models are proposed for comparative analysis to illustrate the great potential of the model in the functional domain, providing a theoretical basis for the effective integration of functional models and the management of environmental pollution problems.

## 2. Model and estimation

### 2.1. Model building

A functional spatial autoregressive combined model is of the form

$$\left\{ \begin{array}{l} y=\rho W_{n}y+\int_{\Gamma }X(t)\beta (t)dt+\mu \\ \mu =\lambda M_{n}\mu +\varepsilon \end{array}.\right.$$ (1)

Let *y* = (*y*_1_,*y*_2_,…,*y*_*n*_)′ be a real-valued random variable defined in the probability space (Ω,ℱ,P), $X(t)=(x_{1}(t),x_{2}(t),\ldots ,x_{n}(t){)}^{\prime }\in L^{2}(\Gamma )$ is a random process independently and identically distributed in the probability space (Ω,ℱ,P), in this paper assume Γ = [0,1], *β*(*t*) is the slope function in the square product, *W*_*n*_ and *M*_*n*_ and is the spatial weight matrix, *ρ* and *λ* is the spatial autoregressive coefficient of the spatial lag term *W*_*n*_*y* and error term *M*_*n*_*μ*, *ε* is the residual term of the model, obeys the multivariate normal distribution *N*(0,*σ*^2^*I*_*n*_).

### 2.2. Maximum likelihood estimation

In this paper, principal component analysis is chosen to achieve the approximation of the slope function. The eigenvalues are arranged in descending order, and the top m principal components whose cumulative variance contribution can reach 85% are selected to truncate the functional part of the model into a simplified scalar form. The model is changed from a functional linear model to an ordinary linear model to achieve the purpose of dimensionality reduction, and the parameter estimation can be further achieved by the maximum likelihood estimation (See S1 Appendix for the specific process).

The above Eq (1) is written as a truncated log-likelihood function of the form is

$$\begin{array}{l} \ln L_{m}=-\frac{n}{2}\ln(2\pi )-\frac{n}{2}\ln\sigma^{2}+\ln|S(\rho )|+\ln|R(\lambda )|\\ \;-\frac{1}{2\sigma^{2}}[S(\rho )Y_{n}-X_{m}\beta_{m}{]}^{\prime }\Omega (\lambda )[S(\rho )Y_{n}-X_{m}\beta_{m}] \end{array}.$$ (2)

Then the partial derivatives of the truncated log-likelihood function ln *L*_*m*_ with respect to *β*_*m*_ and *σ*^2^ is obtained

$$\frac{\partial \ln L_{m}}{\partial \beta_{m}}=X_{m}^{\prime }\Omega (\lambda )[S(\rho )Y_{n}-X_{m}\beta_{m}]=0,$$

$$\frac{\partial \ln L_{m}}{\partial \sigma^{2}}=\frac{-n\sigma^{2}+[S(\rho )Y_{n}-X_{m}\beta_{m}{]}^{\prime }\Omega (\lambda )[S(\rho )Y_{n}-X_{m}\beta_{m}]}{2\sigma^{4}}=0.$$

The maximum likelihood estimates ${\hat{\beta }}_{m}(\rho ,\lambda )$, ${\hat{\sigma }}_{ML}^{2}(\rho ,\lambda )$ of *β* and *σ*^2^, respectively, are calculated as

$${\hat{\beta }}_{m}(\rho ,\lambda )={\left(X_{m}^{\prime }\Omega (\lambda )X_{m}\right)}^{-1}X_{m}^{\prime }\Omega (\lambda )S(\rho )Y_{n},$$ (3)

$${\hat{\sigma }}_{ML}^{2}(\rho ,\lambda )=\frac{1}{n}[S(\rho )Y_{n}-X_{m}\beta_{m}{]}^{\prime }\Omega (\lambda )[S(\rho )Y_{n}-X_{m}\beta_{m}].$$ (4)

Substituting Eqs (3) and (4) into the truncated likelihood function (5), we obtain the following centralized truncated log-likelihood function

$$l_{m}=-\frac{n}{2}-\frac{n}{2}\ln(2\pi )-\frac{n}{2}\ln({\hat{\sigma }}_{ML}^{2}(\rho ,\lambda ))+\ln|I_{n}-\rho W_{n}|+\ln|I_{n}-\lambda W_{n}|.$$ (5)

Since *l*_*m*_ is a highly nonlinear function, we can maximize this function by a numerical optimization algorithm to obtain the estimates of *ρ* and *λ*, $\hat{\rho }$ and $\hat{\lambda }$. And by recomputing ${\hat{\beta }}_{m}(\hat{\rho },\hat{\lambda })$ and ${\hat{\sigma }}_{ML}^{2}(\hat{\rho },\hat{\lambda })$, we obtain the final estimates of *β*_*m*_ and *σ*^2^.

The final estimate of *β*(*t*) is obtained as

$$\hat{\beta }(t)=\sum_{l=1}^{m}{\hat{b}}_{l}{\hat{\varphi }}_{l}(t)={\hat{\beta^{\prime }}}_{m}\Phi (t),$$

where $\Phi (t)=(\varphi_{1}(t),\varphi_{2}(t),\ldots ,\varphi_{m}(t){)}^{\prime }$ is a vector of m functional basis functions.

### 2.3 Model selection

In practical problems, we need to consider the applicability of the model to the data set. As a result, it is important to test spatial correlation before using the model. To select the best functional regression model for the study, we use the global *Moran*′*s I* index in this paper to assess whether the response variable and the error term exhibit spatial autocorrelation. One of the key metrics to measure the spatial correlation between adjacent regions is the *Moran*′*s I* index, which is defined as

$$I=\frac{\sum_{i=1}^{n}\sum_{j=1}^{n}w_{ij}(y_{i}-\bar{y})(y_{j}-\bar{y})}{S^{2}\sum_{i=1}^{n}\sum_{j=1}^{n}w_{ij}}$$

where $S^{2}=\frac{1}{n}\sum_{i=1}^{n}(y_{i}-\bar{y})$, $\bar{y}=\frac{1}{n}\sum_{i=1}^{n}y_{i}$, *y*_*i*_ is the observed value of the *i*th region, and *n* is the total number of regions. When 0<*I*≤1, it means that with the aggregation of spatial distribution locations, the correlation becomes more significant as *I* increases. When −1≤*I*<0, it means that with the dispersion of spatial distribution locations, the correlation becomes more significant as *I* decreases. When *I* = 0, there is no spatial autocorrelation at this time.

The residual test method based on the spatial autoregressive model can determine whether the model contains a spatial lag term. The basic hypothesis is given:

$$\begin{array}{l} H_{0}:residuals\;are\;not\;spatially\;autocorrelated\\ H_{1}:residuals\;are\;spatially\;autocorrelated. \end{array}$$

Under this hypothesis, the test statistic *T* is given as

$$T=\frac{\varepsilon^{\prime }W\varepsilon }{\sigma^{2}[T_{2}-T_{1}^{2}Var(\rho )]}∼\chi^{2}(1),$$

where $T_{1}=tr(MMA^{-1}+M^{\prime }MA^{-1})$, $T_{2}=tr(MM+M^{\prime }M)$, *S* = (*I*−*ρW*), *Var*(*ρ*) is the variance of maximum likelihood estimation of parameters *ρ*. Thus, we could test whether the residuals are spatially correlated.

## 3. Asymptotic properties

In this section, we discuss the asymptotic normality of the parameters *ς* = (*β*,*ρ*,*λ*,*σ*^2^)′ to provide a theoretical basis for further statistical inference. Suppose that *W*_*n*_ = *M*_*n*_, and the following regularity conditions hold:

**Assumption 1:** The elements {*ε*_1_},*i* = 1,…,*n* of *ε* = (*ε*_1_,…,*ε*_*n*_)′ are mutually independent and identically distributed with mean zero and variance *σ*^2^. Its moment *E*(|*ε*|^4+*γ*^) for some *γ*>0 exists.

**Assumption 2:** For any bounded or divergent rate sequence {*h*_*n*_}, the elements {*w*_*n*,*ij*_} of *W*_*n*_ are at most of order $h_{n}^{-1}$, that is $w_{n,ij}=Ο(\frac{1}{h_{n}})$ hold for all *i*, *j*, where for any *i* we have *w*_*n*,*ii*_ = 0.

**Assumption 3:** $\underset{n\to \infty }{\lim}\frac{h_{n}}{n}=0$.

**Assumption 4:** $S_{n}=S(\rho_{0})=I-\rho_{0}W_{n}$ and $R_{n}=R(\lambda_{0})=I-\lambda_{0}W_{n}$ are non-singular matrix.

**Assumption 5:** The row and column sums of the matrix sequences {*W*_*n*_}, {*S*(*ρ*)}, {*R*(*λ*)}, $\left\{S_{n}^{-1}\right\}$ and $\left\{R_{n}^{-1}\right\}$ are consistently bounded.

**Assumption 6:** The row and column sums of the matrices {*S*^2^(*ρ*)} and {*R*^2^(*λ*)} are uniformly bounded.

**Assumption 7:** The elements of *X*_*n*_are consistently bounded, $\underset{n\to \infty }{\lim}\frac{X_{n}^{\prime }X_{n}}{n}$ exists and is non-singular.

**Assumption 8:** The row sums and column sums of {*S*^−1^(*ρ*)} (*ρ*∈P) and {*R*^−1^(*λ*)} (*λ*∈Λ) are consistently bounded, where P is the tight set where the parameters *ρ* are located and Λ is the tight set where the parameters *λ* are located. The truth value *ρ*_0_ is the interior point of P and the truth value *λ*_0_ is the interior point of Λ.

**Assumption 9:** $\underset{n\to \infty }{\lim}\frac{\left(X_{n},G_{n}X_{n}\beta_{0}{)}^{\prime }(X_{n},G_{n}X_{n}\beta_{0}\right)}{n}$ exists and non-singular.

**Theorem 1:** Under Assumptions 1–9, $\sqrt{n}({\hat{\varsigma }}_{n}-\varsigma_{0})\overset{D}{\to }N(0,\Sigma_{\varsigma }^{-1}+\Sigma_{\varsigma }^{-1}\Omega_{\varsigma }\Sigma_{\varsigma }^{-1})$, where $\Omega_{\varsigma }=\underset{n\to \infty }{\lim}\Omega_{\varsigma ,n}$ and $\Sigma_{\varsigma }=-\underset{n\to \infty }{\lim}E(\frac{1}{n}\frac{\partial^{2}\ln L_{m}(\varsigma_{0})}{\partial \varsigma \partial \varsigma^{\prime }})$ exist; if *ε*_*i*_ is normally distributed, then $\sqrt{n}({\hat{\varsigma }}_{n}-\varsigma_{0})\overset{D}{\to }N(0,\Sigma_{\varsigma }^{-1})$.

**Theorem 2:** The confidence band of the slope function *β*(*t*) of 100(1−*α*)% is:

$$[\hat{\beta }(t)-{\hat{\sigma }}_{ML}z_{1-\frac{\alpha }{2}}\sqrt{\Phi^{\prime }(t){\left(X_{m}{}^{\prime }\Omega (\lambda )X_{m}\right)}^{-1}\Phi (t)},\hat{\beta }(t)+{\hat{\sigma }}_{ML}z_{1-\frac{\alpha }{2}}\sqrt{\Phi^{\prime }(t){\left(X_{m}{}^{\prime }\Omega (\lambda )X_{m}\right)}^{-1}\Phi (t)}].$$

## 4. Numerical simulation

The model for calculating the sample simulation data is:

$$Y_{n}={\left(I_{n}-\rho W_{n}\right)}^{-1}[{\int_{\Gamma }X(t)\beta (t)dt+(I_{n}-\lambda M_{n})}^{-1}\varepsilon_{n}].$$

Depending on the different values of the autoregressive parameters *ρ* and *λ*, we consider five cases with different parameters specifically according to the following steps.

1. In this paper, we take four different sample sizes *n* = *R*×*T*, where *R* = (10,20), *T* = (15, 20), R and T represent the number of communities on each side of the study area. According to the different sample sizes, calculate the *n*×*n* row-standardized weight matrices *W*_*n*_ respectively, assuming that *W*_*n*_ = *M*_*n*_.
2. We choose $X(t)=(X_{1}(t),X_{2}(t),\ldots ,X_{n}(t){)}^{\prime },\;i=1,2,\ldots ,n$ from the Brownian motion to model the discrete values of functional covariates, functional regression parameters $\beta (t)=\sqrt{2}\sin(\frac{\pi t}{2})+3\sqrt{2}\sin(\frac{3\pi t}{2})$.
3. We generate a *n*×1 random vectors *ε*_*n*_~*N*(0,*I*_*n*_).
4. In order to evaluate the effect of the spatial coefficients of the model in this paper, five sets of spatial autoregressive coefficients (*ρ*,*λ*) = (0.1,0.9), (*ρ*,*λ*) = (0.3,0.7), (*ρ*,*λ*) = (0.5,0.5), (*ρ*,*λ*) = (0.7,0.3), (*ρ*,*λ*) = (0.9,0.1) and (*ρ*,*λ*) = (0.9,0.1) are considered in this simulation on the simulated sample data y.
5. The parameters *β*(*t*), *ρ*, *λ*, *σ*^2^ are estimated according to the maximum likelihood estimation method procedure described in Section 2.
6. Repeating the Steps 3–5 500 times to calculate the estimates of the parameters ${\hat{\beta }}_{j}(t)$, ${\hat{\rho }}_{j}$, ${\hat{\lambda }}_{j}$, ${\hat{\sigma }}_{j}^{2}$ respectively.

Finally calculate $\hat{\beta }=\frac{1}{500}\sum_{j=1}^{500}{\hat{b}}_{j}{\hat{\varphi }}_{j}(t)$, $\hat{\rho }=\frac{1}{500}\sum_{j=1}^{500}{\hat{\rho }}_{j}$, $\hat{\lambda }=\frac{1}{500}\sum_{j=1}^{500}{\hat{\lambda }}_{j}$, ${\hat{\sigma }}^{2}=\frac{1}{500}\sum_{j=1}^{500}{\hat{\sigma }}^{2}{}_{j}$ and their mean squared errors (MSE).

The results of the simulations of the above steps are presented in Tables 1 and 2 below. The results in both tables show that the procedure of maximum likelihood estimation can accurately estimation of the spatial autocorrelation parameters *ρ* and *λ*. The results in Table 1 show that the estimates are close to the true values for different sample sizes, and the accuracy of the estimates is higher as the sample size increases. The five different spatial autoregressive coefficients’ computed values of ${\hat{\sigma }}^{2}$ are all closer to the real value of 1 when (*ρ*,*λ*) = (0.1,0.9).

**Table 1:** Estimation of spatial autocorrelation coefficients *ρ* and *λ*, and variance *σ*^2^ for different sample sizes (repetitions: 500).

n = R×T	*ρ*	$\hat{\rho }$	*λ*	$\hat{\lambda }$	${\hat{\sigma }}^{2}$
**10×15**	0.1	0.1667	0.9	0.8485	0.9698
	0.3	0.3432	0.7	0.6315	0.9480
	0.5	0.4860	0.5	0.4741	0.9366
	0.7	0.6518	0.3	0.3245	0.9445
	0.9	0.8655	0.1	0.1382	0.9606
**10×20**	0.1	0.1454	0.9	0.8661	0.9768
	0.3	0.3425	0.7	0.6357	0.9593
	0.5	0.4991	0.5	0.4631	0.9468
	0.7	0.6695	0.3	0.3037	0.9514
	0.9	0.8728	0.1	0.1252	0.9665
**20×15**	0.1	0.1258	0.9	0.8807	0.9831
	0.3	0.3191	0.7	0.6685	0.9726
	0.5	0.4881	0.5	0.4897	0.9652
	0.7	0.6677	0.3	0.3209	0.9707
	0.9	0.8809	0.1	0.1238	0.9815
**20×20**	0.1	0.1283	0.9	0.8819	0.9927
	0.3	0.3243	0.7	0.6679	0.9832
	0.5	0.4941	0.5	0.4880	0.9757
	0.7	0.6778	0.3	0.3142	0.9808
	0.9	0.8858	0.1	0.1188	0.9887

**Table 2:** Summary of the estimated effects of the four parameters.

n = R×T	(*ρ*,*λ*)	$MSE(\hat{\rho })$	$MSE(\hat{\lambda })$	$MSE({\hat{\sigma }}^{2})$	$MSE(\hat{\beta }(t))$
**10×15**	(0.1,0.9)	0.0308	0.0145	0.0153	0.6942
	(0.3,0.7)	0.0301	0.0287	0.0162	0.9624
	(0.5,0.5)	0.0229	0.0304	0.0170	0.6894
	(0.7,0.3)	0.0183	0.0355	0.0168	0.6916
	(0.9,0.1)	0.0070	0.0318	0.0161	0.6966
**10×20**	(0.1,0.9)	0.0179	0.0056	0.0122	0.4043
	(0.3,0.7)	0.0263	0.0266	0.0128	0.4055
	(0.5,0.5)	0.0201	0.0317	0.0127	0.4032
	(0.7,0.3)	0.0144	0.0338	0.0124	0.4067
	(0.9,0.1)	0.0060	0.0292	0.0115	0.4140
**20×15**	(0.1,0.9)	0.0132	0.0046	0.0086	0.3105
	(0.3,0.7)	0.0182	0.0142	0.0087	0.3111
	(0.5,0.5)	0.0164	0.0200	0.0083	0.3101
	(0.7,0.3)	0.0129	0.0248	0.0080	0.3125
	(0.9,0.1)	0.0040	0.0184	0.0079	0.3165
**20×20**	(0.1,0.9)	0.0088	0.0021	0.0056	0.3140
	(0.3,0.7)	0.0135	0.0101	0.0055	0.3165
	(0.5,0.5)	0.0117	0.0143	0.0053	0.3161
	(0.7,0.3)	0.0075	0.0150	0.0005	0.3179
	(0.9,0.1)	0.0024	0.0106	0.3220	0.3220

Considering the estimation of the two components of regression parameters and slope function, the mean square error (MSE) is used to measure the goodness of fit of the estimates, and the specific estimation results are evaluated as shown in Table 2. It is found that the MSE of $\hat{\rho }$ is minimum in the case of (*ρ*,*λ*) = (0.9,0.1), while both $\hat{\lambda }$ and $\hat{\beta }(t)$ have the smallest mean square error in the case of (*ρ*,*λ*) = (0.1,0.9) and achieve the best fit. As a whole, the MSE values of the parameters *ρ*, *λ*, and the slope function *β*(*t*) are small, indicating that the fit of each parameter in the model is good; and the fit of the estimates for each sample size in the five cases of (*ρ*,*λ*) gets better as the sample size increases.

**Fig 1:**
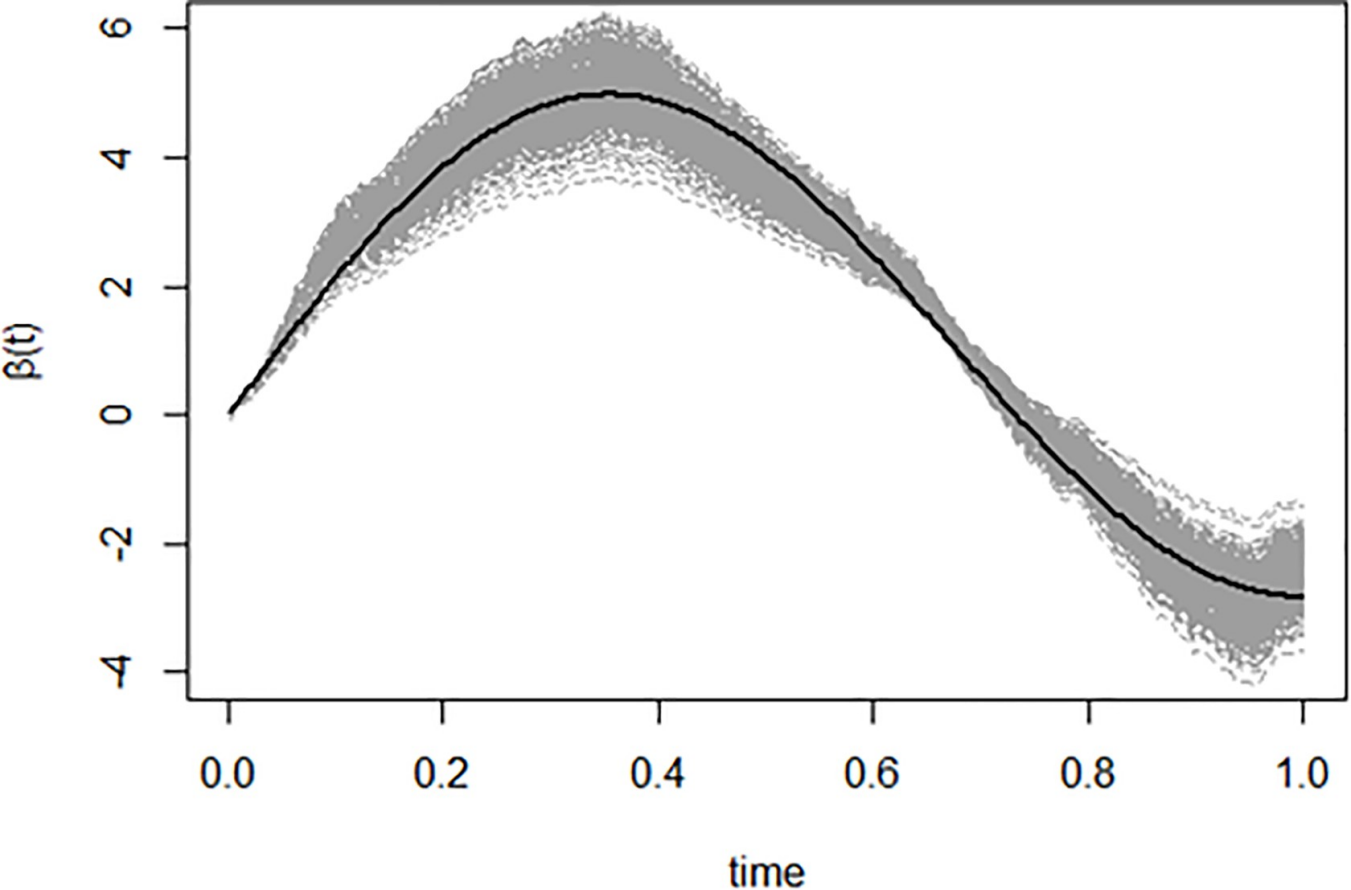
The function parameter *β*(*t*) (black line) and the 500 estimates of this parameter (gray line), where *ρ* = 0.1, *λ* = 0.9.

When *ρ* = 0.1, *λ* = 0.9, in Fig 1 we give 500 estimates of the slope function *β*(*t*), which can prove the effectiveness of the maximum likelihood estimation method.

The empirical confidence intervals and the confidence intervals obtained from the asymptotic distribution of the slope function *β*(*t*) are calculated by Theorem 2 and bootstrap distribution, respectively, and the results are shown in Table 3 below. After 500 iterations of the experiment, we discover that the widths of the confidence intervals obtained by the asymptotic distribution gradually shrink as the sample size increases, and that the interval widths obtained for all four sample sizes are significantly smaller than the interval widths obtained from the empirical confidence intervals, indicating that the properties of the asymptotic distribution obtained in this section have an extremely obvious effect on increasing the precision of the results in our practical applications.

**Table 3:** Interval range of slope function *β*(*t*) for four sample sizes (repetitions: 500).

n = R×T	confidence interval	empirical confidence interval
**10×15**	(-0.1841, 3.8985)	(-2.6505, 4.9414)
**10×20**	(-0.0723, 3.6740)	(-2.7899, 5.2199)
**20×15**	(0.3578, 3.2928)	(-2.6511, 5.0833)
**20×20**	(0.6497, 2.9540)	(-2.8988, 5.1476)

## 5. Functional spatial autoregressive analysis of Fenwei Plain

### 5.1 Data introduction

In this paper, 11 cities in Xi’an, Baoji, Xianyang, Weinan, Tongchuan, Jinzhong, Lvliang, Linfen, Yuncheng, Luoyang, and Sanmenxia were selected as the main study objects in the Fenwei Plain. The Fenwei Plain is a region in the Yellow River Basin with exceptional resource conditions, high levels of industrial and agricultural production, and advanced economic and cultural development. It is distributed in a northeast-southwest direction from Dai County in Shanxi Province to the north, from the Qinling Mountains in Shaanxi Province to the south, and from Baoji City in Shanxi Province to the west. However, the area is vulnerable to the establishment of an anticyclonic airflow stagnation zone due to the blocking effects of mountain ranges and the sinking effect of airflow on the leeward slope. In the face of the severe test of air pollution prevention and control, the prevention and control of air pollution in the Fenwei Plain has become the focus and difficulty of the current environmental quality improvement work. The state and society have paid close attention to the frequent occurrence of air pollution in the Fenwei Plain in recent years, although a study on the circumstances of air quality has primarily concentrated on the eastern region of China, where Beijing, Tianjin, and Hebei are located. However, the Fenwei Plain has seen a sudden increase in pressure to prevent air pollution and the deteriorating ecological environment, but few people have asked about it, so an in-depth study of air pollution in the Fenwei Plain is imperative. From the map of China, we can see that the Fenwei Plain has obvious urban adjacencies geographically, for which we can make a preliminary guess about the spatial correlation of the Fenwei Plain, and the region can be precisely researched using the FSAC model. Therefore, monitoring data of *SO*_2_ concentration (unit: *μg*/*m*3) and time-by-time dew point temperature data (unit:°*C*) for 2019–2021 were collected for detailed analysis in this paper. Data were obtained from the China Air Quality Online Monitoring and Analysis Platform and European Centre for Medium-Range Weather Forecasts (ECMWF).

### 5.2 Data processing and research

**Fig 2:**
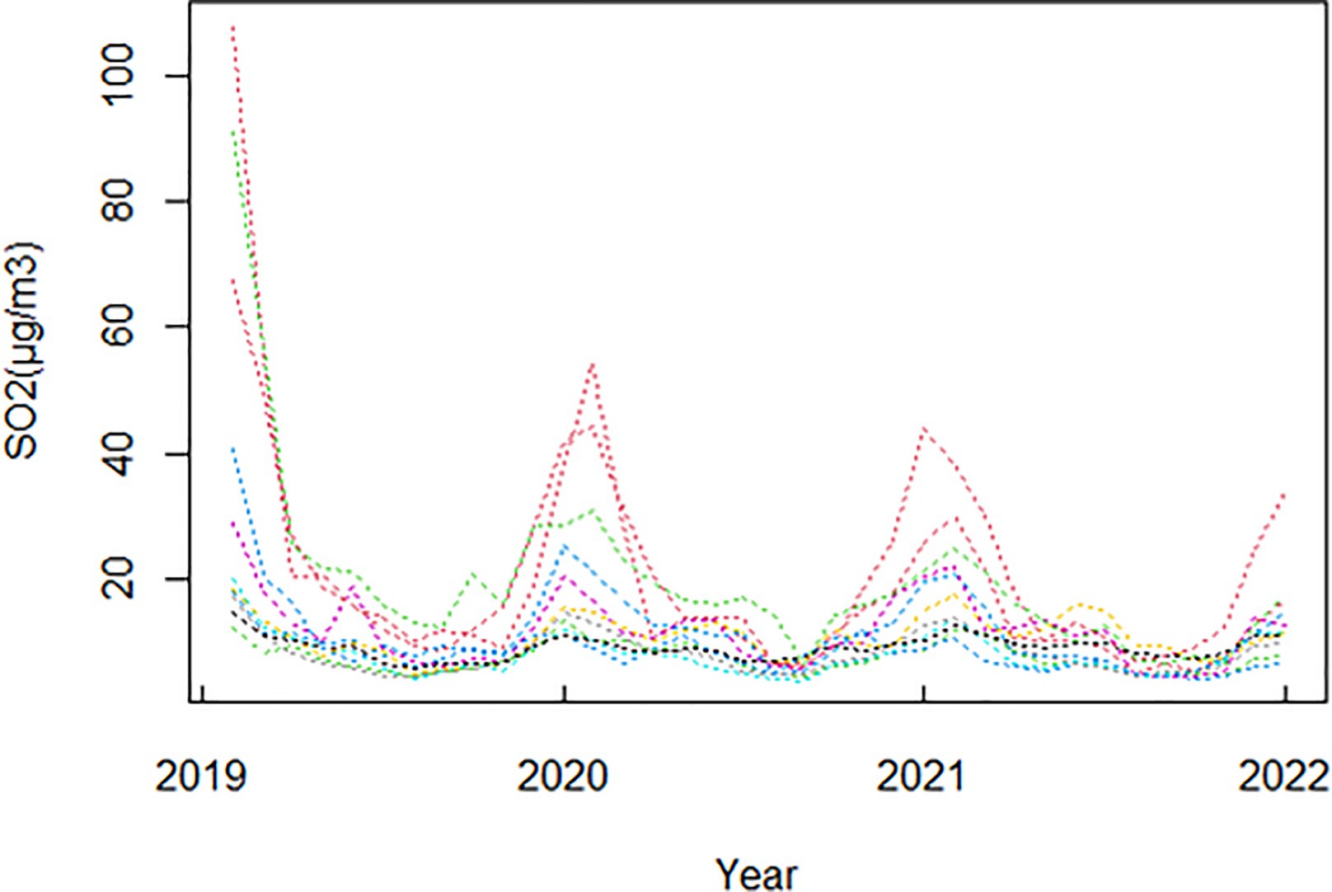
Three-year concentration variation curves for 11 cities in the Fenwei Plain from 2019 to 2021.

The trend of *SO*_2_ concentration changes in 11 cities in the Fenwei Plain from 2019–2021 is shown in Fig 2. We can observe that there is a clear seasonal cycle in the *SO*_2_ concentration, with a clear upward trend at the beginning and end of each year, reaching a peak in winter; as the temperature rises, the *SO*_2_ concentration gradually decreases and reaches its lowest point in summer. This is consistent with the real life, in the northern winter there is a demand for heating, and the main fuel for heating is coal, which will release more pollutants; at the same time, the low temperature, high atmospheric stability and weak solar radiation in the northern winter will cause the *SO*_2_ emissions to become larger and the *SO*_2_ concentration to increase.

In addition, we can see that the *SO*_2_ concentrations in three years are decreasing year by year, which indicates that a series of environmental pollution improvement measures developed by the government has had a significant effect, among which we find a sudden and significant decrease in the concentration in 2020 compared to 2019, which is directly related to the large-scale outbreak of the COVID-19 epidemic in China at the end of 2019, with the passive intervention of the virus, the temporary interruption of production and transportation activities, air pollution improved in various regions of China [30], and *SO*_2_ concentrations decreased significantly.

First, in this paper we choose the global *Moran*′*s I* index to determine whether the *SO*_2_ concentration variable has spatial autocorrelation. The calculated *SO*_2_ concentrations of the 36 months from 2019 to 2021 are shown in Table 4 below.

**Table 4:** Monthly average values of concentrations in 11 cities in the Fenwei Plain from 2019 to 2021.

month	Jan.	Feb.	Mar.	Apr.	May.	Jun.
***Moran*′*s I***	0.5939	0.5471	0.4555	0.5869	0.7190007	0.6070
**P value**	0.0003	0.0011	0.0001	0.0002	0.0000	0.0004
**month**	**Jul.**	**Aug.**	**Sep.**	**Oct.**	**Nov.**	**Dec.**
***Moran*′*s I***	0.5939	0.5471	0.4555	0.5869	0.7197	0.6070
**P value**	0.0003	0.0011	0.0001	0.0002	0.0000	0.0004
**month**	**Jan.**	**Feb.**	**Mar.**	**Apr.**	**May.**	**Jun.**
***Moran*′*s I***	0.5312	0.5954	0.3681	0.3649	0.3759	0.3367
**P value**	0.0007	0.0004	0.0080	0.0129	0.0137	0.0165
**month**	**Jul.**	**Aug.**	**Sep.**	**Oct.**	**Nov.**	**Dec.**
***Moran*′*s I***	0.1182	-0.2119	0.1396	0.5839	0.4155	0.3483
**P value**	0.0432	0.7050	0.1245	0.0005	0.0041	0.0043
**month**	**Jan.**	**Feb.**	**Mar.**	**Apr.**	**May.**	**Jun.**
***Moran*′*s I***	0.6000	0.6258	0.5522	0.3173	0.0588	0.1459
**P value**	0.0002	0.0001	0.0007	0.0265	0.2136	0.1287
**month**	**Jul.**	**Aug.**	**Sep.**	**Oct.**	**Nov.**	**Dec.**
***Moran*′*s I***	-0.2803	-0.1719	-0.0356	-0.1153	0.1837	0.3517
**P value**	0.8052	0.6281	0.03	0.5348	0.0515	0.0014

The findings in Table 4 demonstrate that spatial autocorrelation is not satisfied for more than half a year from May to November in 2021, nor is it satisfied for all 12 months of 2019 or August and September of 2020. We find that the monthly spatial autocorrelation of *SO*_2_ concentrations is weakening year by year, which is partly due to the impact of the emergence of the Novel coronavirus pneumonia. In early 2020, a global outbreak of Novel coronavirus pneumonia began to spread, creating an unprecedented storm in the supply chain of the energy industry worldwide [31]. To stop the spread of COVID-19, China has blocked more than one-third of its cities, and by using difference-in-differences models. After shutting down some of the cities, we can see a sizeable improvement in air pollution [32]. However, other pollution sources persisted in seriously deteriorating local air quality throughout the blockade period, particularly in the northern winter when the government continuously heats residents using a coal-fired central heating system, which produces significant amounts of the same emissions. Therefore, despite the improvement in air quality during the epidemic, air pollution in the Fenwei Plain is still relatively severe in winter. And as the weather warms up, the demand for coal decreases after the cessation of heating, and the impact of sealing the control cities on better air quality becomes more pronounced [33]. Meanwhile, with the successful conclusion of the “The Three-Year Action Plan to Fight Air Pollution” in 2021, the total emissions of major air pollutants are significantly reduced and the ambient air quality improves significantly, which in turn affects the spatial autocorrelation of *SO*_2_ concentration and the impact expands for two consecutive years. From the above results, this paper can select the data of January-April and November-December 2021 for further judgment, and the spatial autocorrelation of *SO*_2_ concentrations in these six months is very significant, the p-value is much less than 0.05.

The data were tested according to the residual test method proposed in the model selection process, and the results are shown in Table 5 below: the model residuals for the six months considered above are spatially autocorrelated, except for January, where the p-value of the residuals is not significant.

**Table 5:** Residual test results based on FSAC model.

month	Jan.	Feb.	Mar.	Apr.	May.	Jun.
**Test value**	9.24×10^−3^	-1.13×10^−3^	4.96×10^−4^	-1.52×10^−3^	-5.44×10^−3^	-9.44×10^−3^
**P value**	0.1531	0	0.0356	0	0	0
**month**	**Jul.**	**Aug.**	**Sep.**	**Oct.**	**Nov.**	**Dec.**
**Test value**	-7.15×10^−3^	-3.90×10^−3^	-5.26×10^−3^	-4.40×10^−3^	-7.72×10^−4^	-8.70×10^−3^
**P value**	0	0	0	0	0	0

### 5.3 Model comparison

In this section, we compare the results of four models, i.e., models (6)-(9), model (6) is an ordinary functional linear model, model (7) is a functional simultaneous autoregressive model with the introduction of a spatial error term, model (8) is a functional spatial autoregressive model with the introduction of a spatial lag term, and Model (9) is the FSAC model with both a spatial lag term and a spatial error term that is the main study in this paper.


$$FLM:\;y=\beta_{0}+\int_{\Gamma }x(t)\beta (t)dt+\varepsilon ,$$ (6)

$$FSAR(1):\;\{\begin{array}{l} y=\int_{\Gamma }x(t)\beta (t)dt+\mu \\ \mu =\lambda M\mu +\varepsilon \end{array},$$ (7)

$$FSAR(2):\;y=\rho Wy+\int_{\Gamma }x(t)\beta (t)dt+\varepsilon ,$$ (8)

$$FSAC:\;\{\begin{array}{l} y=\rho Wy+\int_{\Gamma }x(t)\beta (t)dt+\mu \\ \mu =\lambda M\mu +\varepsilon \end{array},$$ (9)

The mean squared error (MSE) can be used to evaluate the model performance, so we calculated the MSE values of the four models separately as shown in Table 5 below, and analyzed the models month by month: from Tables 4 and 5, we know that the *SO*_2_ concentration in January satisfies the spatial autocorrelation, but the residual test does not pass, so the MSE of the models (8) and (9) with the addition of the spatial lag term is more advantageous than that of models (6) and (7) by fitting the above four function-based models. However, for the functional spatial linear model (6) with the addition of the insignificant spatial error term, the fit is not good and the MSE is much larger than that of the other three models. The situation is similar for March, April and December, and the above results verify the existence of spatial autocorrelation of the residuals along with the existence of spatial autocorrelation of the *SO*_2_ concentrations in these three months. Therefore, the results from Table 6 show that adding either the spatial lag term or the spatial error term on top of the functional linear model (6) is better for the simulation of the model itself, and the MSE is reduced to some extent, and the MSE value of model (9) is the smallest and the best among the four models. February is the most special month in our study, and Table 6 shows that although February satisfies the spatial autocorrelation of *SO*_2_ concentrations and the autocorrelation of residuals as well as the other months, and all the other three models improve their fits on the basis of model (6), the MSE of model (9) is worse than that reflected by models (7) and (8).

**Table 6:** MSE values of the four functional spatial models.

month	January	February	March	April	December
**MSE(FLM)**	0.8932	1.5451	0.4422	2.1964	3.2191
**MSE(FSAR1)**	5.2384	1.0808	0.2822	2.0911	0.7069
**MSE(FSAR2)**	0.5955	1.3096	0.0993	1.8450	2.4829
**MSE(FSAC)**	0.7444	1.4931	0.0784	1.5267	0.6323

It is also observed that the MSE of the FLM model increases significantly in December, which is partly due to the human influence of winter heating in December in the north, while the MSE of the FSAC model is much lower than that of the FLM model in that month, which indicates that the FSAC model reduces the human influence. In summary, it can be proved that the FSAC model has a better fitting effect when dealing with data with spatial autocorrelation properties.

### 5.4 Functional spatial autoregressive analysis

Firstly, the model is truncated by using functional principal component analysis. The first four principal components are extracted from the original data of 11 cities in Fenwei Plain, and it is found that the cumulative contribution of the first four principal components has reached 85%, so the first four principal components can be selected to reduce the dimensionality of the model and realize the simplification process from the infinite-dimensional functional spatial autoregressive model

$$\left\{ \begin{array}{l} y_{i}=\rho \sum_{j=1}^{11}w_{ij}y_{j}+\int_{\Gamma }x_{i}(t)\beta (t)dt+\mu_{i}\\ \mu_{i}=\lambda \sum_{j=1}^{11}w_{ij}\mu_{j}+\varepsilon_{i} \end{array},\;i=1,\ldots ,11\right.$$

to the spatial autoregressive model

$$\left\{ \begin{array}{l} y_{i}=\rho \sum_{j=1}^{11}w_{ij}y_{j}+\sum_{j=1}^{4}a_{ij}b_{j}+\mu_{i}\\ \mu_{i}=\lambda \sum_{j=1}^{11}w_{ij}\mu_{j}+\varepsilon_{i} \end{array},\;i=1,\ldots ,11.\right.$$

**Fig 3:**
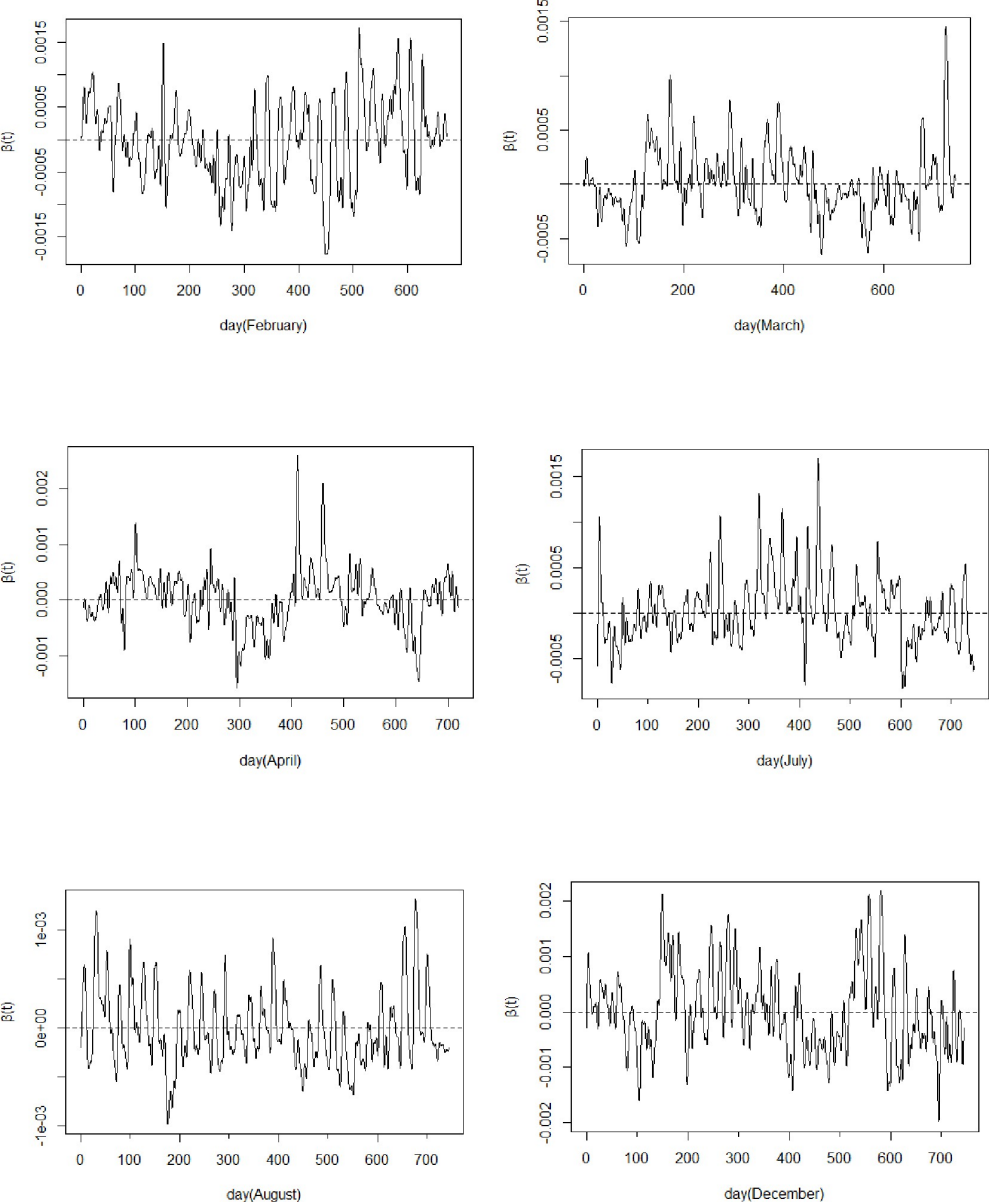
Slope function *β*(*t*) image of FSAC model for six months.

The FSAC model was used to estimate the slope function *β*(*t*) for the five months selected in previous section. The estimated results of the slope function *β*(*t*) are shown in Fig 3 below. From Fig 3, it can be seen that the negative range of February is significantly larger than the positive value, indicating that the effect of dew point temperature on the *SO*_2_ concentration is somewhat negatively correlated in this month, and the negative value of *β*(*t*) has a significant tendency to become larger during the 2021 Chinese New Year period, i.e. 200–300 days, indicating that during the Chinese New Year period due to the fireworks and the increase in fuel use will have a certain effect on the *SO*_2_ concentration, thus making the effect of dew point temperature on the *SO*_2_ concentration more disturbed, but the impact can be deceptive. The range of *β*(*t*) values in August shows a relatively uniform symmetry, there is no obvious positive or negative tendency in the correlation between dew point temperature and *SO*_2_ concentration in that month. While the range of positive values for the other months in this paper is obviously more than the range of negative values, this indicates that the dew point temperature has a greater positive influence on the concentration, i.e., when the temperature increases, the *SO*_2_ concentration is likely to increase.

## 6. Conclusion

The main work of this paper is to combine the functional-type linear model with the spatial autoregressive model, to extend the functional model to the functional spatial autoregressive combined model, and to improve the estimation theory of both the parameter estimation and the large sample characteristics of this model. Firstly, the FSAC model uses functional principal component analysis to truncate the functional part, transforming the original functional model into a linear model with scalar covariates, and then using the maximum likelihood estimation can be used to obtain the parameter estimates. In addition, we can prove the asymptotic normality of the parameter estimate after giving certain regularity conditions. Subsequently, we design a simulation test to verify the validity of the model. Five hundred simulations were conducted with five different parameters (*ρ*,*λ*) and four sample sizes. The results proved that the estimation results obtained in various cases were different, still all simulations performed better, and the asymptotic distributions obtained were more effective for the calculation of confidence intervals for the slope functions. Finally, the FSAC model was applied to a specific study of weather pollution in the Fenwei Plain, and the results are summarized below.

1. The spatial autocorrelation of the three-year *SO*_2_ concentration *Moran*′*s I* results shows that the spatial autocorrelation decreases year by year, and is more obvious in the summer when the temperature is higher, the *SO*_2_ concentration is lower, and the pollutant emission is less. This indicates that a series of policies implemented by the government have a very significant effect on the improvement of environmental pollution in the Fenwei Plain.
2. Both *SO*_2_ concentration and temperature data have seasonal periodicity, so we can select a certain number of Fourier basis functions for the model to reduce the dimensionality of the functional variables. However, since January or February each year will be influenced by the biggest festival in China—Chinese New Year, the empirical results are highly likely to be biased by external interference. They should be considered in the context of the actual situation.
3. It is shown that the FSAR model and FSAC model can depict the spatial autocorrelation of monthly concentrations more accurately in the Fenwei Plain compared with the functional linear model. And when comparing the MSE of the FSAC model with FLM and two FSAR models, it is discovered that the FSAC model can significantly improve the model fitting efficiency and lessen the impact of human errors under the actual circumstance that both dependent variables and error terms satisfy spatial autocorrelation in this paper.
4. The functional spatial autoregressive combination model is chosen to deal with high frequency and high density of actual observation data, which can analyze the effect of temperature change on *SO*_2_ concentration not only at the macroscopic level throughout the year but also at the microscopic level where the effect of hourly temperature data change on *SO*_2_ concentration is obvious. Then it can be effectively applied to environmental control and monitoring as a new technology.
5. For this paper, there are still some limitations and problems to be further studied. Firstly, in terms of data pre-processing, the Fourier basis function is used for data dimensionality reduction when the observed data are functionalized, which can be used for regular data. But in the face of the observed data without special rules, the method of fitting is no longer applicable. Secondly, only a set of functional covariates is selected in this paper, and other weather-related covariates can be added for further study in the future.

## Supporting information

S1 Appendix Proof of extremely large likelihood estimation. (PDF)

S2 Appendix Proof of asymptotic properties. (PDF)

## References

[1] Chan CK, Yao X. Air pollution in mega cities in China. Atmos. Environ. 2008;42:1–42. 10.1016/j.atmosenv.2007.09.003

[2] Niu Y, Chen RJ, Kan HD. Air pollution, disease burden, and health economic loss in China. Ambient Air Pollution and Health Impact in China. 2017;1017:233–242. 10.1007/978-981-10-5657-4_10 29177965

[3] Wei YG, Gu J, Wang HW, Yao T, Wu ZZ. Uncovering the culprits of air pollution: Evidence from China’s economic sectors and regional heterogeneities. Journal of Cleaner Production. 2018;171:1481–1493. 10.1016/j.jclepro.2017.09.246

[4] Jia RX, Ku HJ. Is China’s pollution the culprit for the choking of South Korea? Evidence from the Asian Dust. Econ. J.2019;129:3154–3188. 10.1093/ej/uez021

[5] Li F, Zhou T. Effects of urban form on air quality in China: An analysis based on the spatial autoregressive model. Cities. 2019;89:130–140. 10.1016/j.cities.2019.01.025

[6] Chen CQ, Chen YQ, Tang SJ, Wu SJ. Analysis of effect of meteorological factor on air quality of Wuhan in 2001–2010. Environmental Science and Technology.2013;36(5): 130–133.

[7] Huang JT. Sulfur dioxide (SO2) emissions and government spending on environmental protection in China—Evidence from spatial econometric analysis. J. Clean. Prod. 2018;175:431–441. 10.1016/j.jclepro.2017.12.001

[8] Lou LY, Li J, Zhong S. Sulfur dioxide (SO2) emission reduction and its spatial spillover effect in high-tech industries: based on panel data from 30 provinces in China. Environmental Science and Pollution Research. 2021;28(24):31340–31357. 10.1007/s11356-021-12755-7 33604830

[9] Jiang L, He SX, Cui YZ, Zhou HF, Kong H. Effects of the socio-economic influencing factors on SO2 pollution in Chinese cities: a spatial econometric analysis based on satellite observed data. J. Environ. Manag. 2020;268. 10.1016/j.jenvman.2020.110667 32383661

[10] Li R, Fu HB, Cui LL, Li JL, Wu Y, Meng Y, et al. The spatiotemporal variation and key factors of SO2 in 336 cities across China. J. Clean. Prod. 2019;210:602–611. 10.1016/j.jclepro.2018.11.062

[11] Hassan MH, Mostafa SA, Mustapha A, Saringat M, Alrimy BAS, Saeed F, et al. A new collaborative multi-agent Monte Carlo simulation model for spatial correlation of air pollution global risk assessment. Sustainability. 2020;14(1). 10.3390/su14010510

[12] Jiang J, Zha Y, Li L. Simulation analysis of atmospheric SO2 contributions from different regions in China. Atmospheric Pollution Research. 2019;10(3):913–920. 10.1016/j.apr.2018.12.019

[13] Xu DD, Wang JT, Yuan ZB, Huang JP, Zhao KH. Temporal-spatial variations, source apportionment, and formation mechanisms of PM_(2.5) pollution over Fenwei Plain, China. Acta Scientiae Circumstantiae. 2021;41(04):1184–1198. 10.13671/j.hjkxxb.2020.0553

[14] Li YY, Li L, Zeng SL, Wang W. Analysis of atmospheric particulates in the eastern Fenwei Plain in 2007. Research of Environmental Sciences. 2019;33(01):63–72. doi: 10.13198/j.issn.1001-6929.2019.06.16

[15] Zhang HY, Di BF, Liu DR, Li JR, Zhan Y. Spatiotemporal distributions of ambient SO2 across China based on satellite retrievals and ground observations: Substantial decrease in human exposure during 2013–2016. Environmental Research. 2019;179. doi: 10.1016/j.envres.2019.108795 31605867

[16] Wang YQ, Xu K, Li SM. The Functional spatio-temporal statistical model with application to O-3 pollution in Beijing, China. International Journal of Environmental Research and Public Health. 2020;17(9). doi: 10.3390/ijerph17093172 32370183 PMC7246770

[17] Li YY, Li J, Yang WY, Wang LL, Ma SL. Transport characteristics of atmospheric particulates in Fenwei Plain and its surrounding regions in 2018. Acta Scientiae Circumstantiae.2020;40(3):779–791. 10.13671/j.hjkxxb.2019.0417

[18] Zhan DS, Kwan MP, Zhang WZ, Yu XF, Meng B, Liu QQ. The driving factors of air quality index in China. Journal ofleaner Prod Cuction. 2018;197:1342–1351. 10.1016/j.jclepro.2018.06.108

[19] Wei W, Wang LJ, Jin ZH, Chen X, Zhang YJ. The spatio-temporal distribution characteristics of atmospheric SO_2 in Fenwei Plain based on OMI data. Ecology and Environmental Sciences. 2018;27(12):2276–2283. 10.16258/j.cnki.1674-5906.2018.12.013.

[20] Zhang YJ, Zhu LY, Guo W, Zhou J, Han ZY, Li F, et al. Analysis of temporal and spatial variation characteristics of atmospheric SO_2 and NO_2 in Fenwei Plain. Ecology and Environmental Sciences. 2020;29(6):1147–1156. 10.16258/j.cnki.1674-5906.2020.06.010

[21] Hao YP, Song XW, Zhao WJ, Xiang FM. Spatiotemporal distribution of air pollution and correlation factors in Fenwei Plain. Ecology and Environmental Sciences. 2022;31(03):512–523. 10.16258/j.cnki.1674-5906.2022.03.010

[22] Zheng XH, Li MX, Liu H, Lou PH. Spatiotemporal characteristics of air quality and their relations meteorological factors over the Fenwei Plain. Acta Scientiae Circumstantiae. 2020;40(11):4113–4121. 10.13671/j.hjkxxb

[23] Ramsay JO, Dalzell CJ. Some tools for functional data analysis. Journal of the Royal Statistical Society: Series B(Methodological). 1991;53(3):539–561.

[24] Ramsay JO, Silverman BW. Functional data analysis (Second Edition). New York: Springer. 2005.

[25] Ahmed MS. Contribution to spatial statistics and functional data analysis. Lille: Universite Charles de Gaulle-Lille Ⅲ. 2017.

[26] Pineda-Ríos W, Giraldo R, Porcu E. Functional SAR models: With application to spatial econometrics. Spatial Statistics. 2018;29:145–159. 10.1016/j.spasta.2018.12.002

[27] Aw A, Cabral EN. Functional SAC model: With application to spatial econometrics. South African Statistical Journal. 2021;55(1):1–13.

[28] Martinez J, Saavedra A, Garcia-Nieto PJ et al. Air quality parameters outliers detection using functional data analysis in the Langreo urban area (Northern Spain). Applied mathematics and computation. 2014;241:1–10. 10.1016/j.amc.2014.05.004

[29] Hu XJ, Li YL, Shi XP. Functional spatial autoregressive analysis of SO2 and air temperature in Fenwei Plain. Science Technology and Engineering. 2021;21(28):11938–11946.

[30] Gan WT, Zhou MY, Cheng YR, Ye L, Chen J, Wang MW, et al. Air quality and the COVID-19 outbreak in China. Allergologia et immunopathologia. 2021;49(1):165–167. doi: 10.15586/aei.v49i1.66 33528946

[31] Wang QR. The influence of COVID-19 epidemic on Shanxi coal industry and relevant Suggestions. China Coal. 2020;46(05):7–9. 10.19880/j.cnki.ccm.2020.05.001

[32] He GJ, Pan YH, Tanaka T. The short-term impacts of COVID-19 lockdown on urban air pollution in China. Nature Sustainability. 2020;3(12). 10.1038/s41893-020-0581-y

[33] Fan MY, He GJ, Zhou MG. The winter choke: coal-fired heating, air pollution, and mortality in China. Health Econ. 2020;71. doi: 10.1016/j.jhealeco.2020.102316 32179329

