# Supplementary material for: Analysis of air pollution in Fenwei Plain in China based on functional spatial autoregressive combined model
Source: PLoS One. 2023 May 12;18(5):e0283336. doi: 10.1371/journal.pone.0283336 (PMC10180685; doi:10.1371/journal.pone.0283336)
Supplement: S1 Appendix — (PDF) [file pone.0283336.s001.pdf]

S1 Appendix. Proof of maximum likelihood estimation.

We know that the covariance function of $X(t)$ is

$$k(s,t) = \mathrm{cov}(X(s), X(t)),$$

consider the empirical covariance function of $X(t) = (x_1(t), x_2(t), \cdots, x_n(t))'$, which is given by

$$\hat{k}(s,t) = \sum_{i=1}^{\infty} X_i(s) X_i(t).$$

According to Mercer's theorem, we obtain the spectral decomposition of $\hat{k}(s,t)$

$$\hat{k}(s,t) = \sum_{i=1}^{\infty} X_i(s) X_i(t) = \sum_{j=1}^{\infty} \hat{k}_j \hat{\varphi}_j(s) \hat{\varphi}_j'(t),$$

where $\hat{k}_1 > \hat{k}_2 > \cdots > 0$ is the eigenvalue of $\hat{k}(s,t)$, and $\{\hat{\varphi}_j(t)\}$ is its corresponding sequence of standard orthogonal eigenfunctions. From the Karhunen-Loève theorem, we know that

$$X(t) = \sum_{j=1}^{\infty} a_j \varphi_j(t), \quad \beta(t) = \sum_{l=1}^{\infty} b_l \varphi_l(t),$$

where $a_j = \int_\Gamma X(t)\varphi_j(t)dt$ and $b_l = \int_\Gamma \beta(t)\varphi_l(t)dt$ are the jth functional principal component score of $X(\cdot)$ and the lth functional principal component score of $\beta(\cdot)$, respectively, both $\{a_j\}$ and $\{b_l\}$ are uncorrelated random variables, and $a_j = (a_{1j}, a_{2j}, \cdots, a_{nj})'$ satisfy $Ea_j = 0$, $\mathrm{var}(a_j) = k_j$ for $j = 1, \cdots, m$. Then for the sample observations the estimates are $x_i(t) = \sum_{j=1}^{\infty} \hat{a}_{ij} \hat{\varphi}_j(t)$, where $\hat{a}_j = \int_\Gamma X(t)\hat{\varphi}_j(t)dt$. Similarly, $\beta(t) = \sum_{j=1}^{\infty} \hat{b}_j \hat{\varphi}_j(t)$, where $\hat{b}_j = \int_\Gamma \beta(t)\hat{\varphi}_j(t)dt$.

In summary based on the orthogonality of $\{\hat{\varphi}_j(t)\}$, there are equivalent expressions

$$\int_\Gamma X(t)\beta(t)dt = \sum_{j=1}^{\infty} a_j b_j = \sum_{j=1}^{m} a_j b_j + \sum_{j=m+1}^{\infty} a_j b_j ,$$

where m is the truncation parameter, $m \le n$, and m can be chosen by methods such as the Bayesian information criterion (BIC). After dimensionality reduction by the above principal components, the part of function-type variable can be approximated as

$$\int_\Gamma X(t)\beta(t)dt = \sum_{j=1}^{m} a_j b_j ,$$

where m is the number of principal components that reach 85% of the variance contribution. Let $x_m = (a_{ij})_{n\times m}$, $\beta_m = (b_1, b_2, \cdots, b_m)'$, then the truncation form of the above equation is

$$\int_\Gamma X(t)\beta(t)dt = X_m \beta_m . \tag{A.1}$$

Next, the maximum likelihood estimation of the unknown parameters is performed, according to (1) we have

$$\begin{cases} (I_n - \rho W_n)Y_n = \int_\Gamma X(t)\beta(t)dt + \mu_n \\ (I_n - \lambda W_n)\mu_n = \varepsilon_n \end{cases} . \tag{A.2}$$

Let $S(\rho) = I_n - \rho W_n$, $R(\lambda) = I_n - \lambda M_n$, by simplifying we obtain

$$\varepsilon = R(\lambda)[S(\rho)Y_n - \int_\Gamma X(t)\beta(t)dt] . \tag{A.3}$$

Since $\varepsilon \sim N(0, \sigma^2 I_n)$, the probability density function of is given by

$$f(\varepsilon \mid \beta, \rho, \lambda, \sigma^2) = (2\pi)^{-\frac{n}{2}} (\sigma^2)^{-\frac{n}{2}} \exp\{-\frac{\varepsilon^T \varepsilon}{2\sigma^2}\} ,$$

where $\varepsilon^T \varepsilon = [S(\rho)Y_n - \int_\Gamma X(t)\beta(t)dt]' R'(\lambda) R(\lambda) [S(\rho)Y_n - \int_\Gamma X(t)\beta(t)dt]$.

According to the transformation theorem, the probability density function of Y_n is

$$f(Y_n \mid \beta, \rho, \lambda, \sigma^2) = (2\pi)^{-\frac{n}{2}} (\sigma^2)^{-\frac{n}{2}} \exp\{-\frac{\varepsilon^T \varepsilon}{2\sigma^2}\} \left| \frac{\partial \varepsilon}{\partial Y_n} \right| = (2\pi)^{-\frac{n}{2}} (\sigma^2)^{-\frac{n}{2}} |S(\rho)| |R(\lambda)| \exp\{-\frac{\varepsilon^T \varepsilon}{2\sigma^2}\},$$

where $\left| \frac{\partial \varepsilon}{\partial Y_n} \right|$ is a Jacobi determinant.

Let $\Omega(\lambda) = R'(\lambda) R(\lambda) = (I_n - \lambda M_n)'(I_n - \lambda M_n)$, and the likelihood function is

$$L = (2\pi)^{-\frac{n}{2}} (\sigma^2)^{-\frac{n}{2}} |S(\rho)| |R(\lambda)| \exp\{-\frac{1}{2\sigma^2}[S(\rho)Y_n - \int_\Gamma X(t)\beta(t)dt]' \Omega(\lambda) [S(\rho)Y_n - \int_\Gamma X(t)\beta(t)dt]\} \cdot$$

The expression of the log-likelihood function is given by

$$\begin{aligned} \ln L = & -\frac{n}{2}\ln(2\pi) - \frac{n}{2}\ln\sigma^2 + \ln|S(\rho)| + \ln|R(\lambda)| \\ & -\frac{1}{2\sigma^2}[S(\rho)Y_n - \int_\Gamma X(t)\beta(t)dt]' \Omega(\lambda) [S(\rho)Y_n - \int_\Gamma X(t)\beta(t)dt]. \end{aligned}$$
