# Supplementary material for: Analysis of air pollution in Fenwei Plain in China based on functional spatial autoregressive combined model
Source: PLoS One. 2023 May 12;18(5):e0283336. doi: 10.1371/journal.pone.0283336 (PMC10180685; doi:10.1371/journal.pone.0283336)
Supplement: S2 Appendix — (PDF) [file pone.0283336.s002.pdf]

S2 Appendix. Proof of asymptotic properties.

Lemma 1: Suppose that the elements $a_{n,ij}$ of a $n \times n$ matrix A_n are $\mathrm{O}(\frac{1}{h_n})$ for all *i, j*. If there exists a $n \times n$ matrices $\{B_n\}$ whose column sums (or row sums) are consistently bounded, then the elements of $A_n B_n$ ($B_n A_n$) have uniform order $\mathrm{O}(\frac{1}{h_n})$. In this case, there is $tr(A_n B_n) = tr(B_n A_n) = \mathrm{O}(\frac{n}{h_n})$.

Lemma 2: Suppose that the row sum or column sum of $\{A_n\}$ is consistently bounded and its elements exist $\mathrm{O}(\frac{1}{h_n})$ in *i* and *j*. Then there is $E(V_n' A_n V_n) = \mathrm{O}(\frac{n}{h_n})$, $\mathrm{var}(V_n' A_n V_n) = \mathrm{O}(\frac{n}{h_n})$; if $\lim_{n\to\infty}(\frac{h_n}{n}) = 0$, then $\frac{h_n}{n}[V_n' A_n V_n - E(V_n' A_n V_n)] = o_p(1)$.

Lemma 3: Suppose that is a square matrix A_n with uniformly bounded column sums, and all elements of the $n \times m$ matrix Z_n are uniformly bounded, then $(\frac{1}{\sqrt{n}}) Z_n' A_n V_n = \mathrm{O}_P(1)$. Also, if $\frac{Z_n' A_n A_n' Z_n}{n} = \mathrm{O}_P(1)$ exists and is positive definite, then $(\frac{1}{\sqrt{n}}) Z_n' A_n V_n \xrightarrow{D} N(0, \sigma_0^2 \lim_{n\to\infty} \frac{Z_n' A_n A_n' Z_n}{n})$

(Lung-Fei Lee, 2004).

Proof of Theorem 1: Let $V_n = \varepsilon_n = R(\lambda)[S^{-1}(\rho) Y_n - X_m \beta_m]$, $X_m^* = R(\lambda) X_m$. The asymptotic distribution of $V_n = \varepsilon_n = R(\lambda)[S^{-1}(\rho) Y_n - X_m \beta_m]$, $X_m^* = R(\lambda) X_m$. $\hat{\varsigma}_n = (\hat{\beta}_n, \hat{\rho}_n, \hat{\lambda}_n, \hat{\sigma}_n^2)'$ can be obtained from the Taylor expansion in that the first-order derivative of the log-likelihood function has the following value at

$$\frac{\partial \ln L_m(\varsigma_0)}{\partial \beta} = \frac{1}{\sigma^2}(X_m^*) V_n, \tag{A.4}$$

$$\frac{\partial \ln L_m(\varsigma_0)}{\partial \rho} = \frac{1}{\sigma^2}(R(\lambda) G_n X_m \beta_m)' V_n + \frac{1}{\sigma^2}(V_n' R^{-1}(\lambda) G_n R(\lambda) V_n - \sigma^2 tr(G_n)), \tag{A.5}$$

$$\frac{\partial \ln L_m(\varsigma_0)}{\partial \lambda} = \frac{1}{\sigma^2}(V_n' F_n V_n - \sigma^2 tr(F_n)), \tag{A.6}$$

$$\frac{\partial \ln L_m(\varsigma_0)}{\partial \sigma^2} = \frac{1}{2\sigma^4}(V_n' V_n - n\sigma^2), \tag{A7}$$

where $G_n = W_n S_n^{-1}$, $F_n = W_n R_n^{-1}$, the asymptotic distribution of (3.2) can be obtained by the central limit theorem for linear quadratic functions. When $\{h_n\}$ is a bounded sequence, the central limit

theorem of the linear quadratic form proposed by Kelejian and Prucha [34] applies; when $\lim_{n\to\infty} h_n = \infty$,

$$\mathrm{var}(\frac{1}{\sqrt{n}} V_n' R^{-1}(\lambda) G_n R(\lambda) V_n) = \mathrm{O}(\frac{1}{h_n}),$$

and it follows from Lemma 2 and Lemma 3 that

$$\frac{1}{\sqrt{n}}(V'(\tau) R^{-1}(\lambda) G_n R(\lambda) V(\tau) - \sigma^2 tr(G_n)) = o_P(1),$$

$$\frac{1}{\sqrt{n}}(V_n' G_n V_n - \sigma_0^2 tr(G_n)) = \mathrm{O}_P(1).$$

Therefore, this term $\frac{1}{\sqrt{n\sigma^2}}(R(\lambda) G_n X_m \beta_m)' V_n$ will dominate the results in the case $\frac{1}{\sqrt{n}} \frac{\partial \ln L_m(\varsigma_0)}{\partial \rho}$ where Kolmogorov's central limit theorem is applicable.

The covariance information array of $\frac{1}{\sqrt{n}} \frac{\partial \ln L_m(\varsigma_0)}{\partial \varsigma}$ is:

$$E(\frac{1}{\sqrt{n}} \frac{\partial \ln L_m(\varsigma_0)}{\partial \varsigma} \cdot \frac{1}{\sqrt{n}} \frac{\partial \ln L_m(\varsigma_0)}{\partial \varsigma}) = -E(\frac{1}{n} \frac{\partial^2 \ln L_m(\varsigma_0)}{\partial \varsigma \partial \varsigma'}) + \Omega_{\varsigma,n},$$

where

$$-E(\frac{1}{n} \frac{\partial^2 \ln L_m(\varsigma_0)}{\partial \varsigma \partial \varsigma'})$$
$$= \begin{pmatrix} 0 & 0 & 0 & 0 \\ 0 & \frac{1}{n} tr(G_n^s G_n) & 0 & 0 \\ 0 & 0 & 0 & 0 \\ 0 & 0 & 0 & 0 \end{pmatrix} +$$
$$\begin{pmatrix} \frac{1}{n\sigma_0^2}(X_m^*)' X_m^* & \frac{1}{n\sigma_0^2}(X_m^*)' R(\lambda_0) G_n X_m \beta_m & 0 & 0 \\ \frac{1}{n\sigma_0^2}(R(\lambda_0) G_n X_m \beta_m)' X_m^* & \frac{1}{n\sigma_0^2}(R(\lambda_0) G_n X_m \beta_m)' R(\lambda_0) G_n X_m \beta_m & \frac{1}{n} tr(G_n F_n^s) & \frac{1}{n\sigma_0^2} tr(G_n) \\ 0 & \frac{1}{n} tr(G_n F_n^s) & \frac{1}{n} tr(F_n^s F_n) & \frac{1}{n\sigma_0^2} tr(F_n) \\ 0 & \frac{1}{n} tr(G_n) & \frac{1}{2n\sigma_0^2} tr(F_n) & \frac{2-n}{2\sigma_0^4} \end{pmatrix},$$

$G_n^s = G_n + G_n'$ and $F_n^s = F_n + F_n'$ are both mean Hessian matrices (information arrays when ε subject to normal distribution).

$$\Omega_{\varsigma,n} = \begin{pmatrix} 0 & 0 & 0 & 0 \\ 0 & \frac{\mu_3}{n\sigma_0^4} R^{-1}(\lambda_0)G_n R(\lambda_0)(R(\lambda_0)G_n X_m \beta_m) + \frac{\mu_4 - 3\sigma_0^4}{n\sigma_0^4} tr(G_n^2) & * & * \\ 0 & \frac{\mu_3}{n\sigma_0^4} F_n R(\lambda_0)G_n X_m \beta_m + \frac{\mu_4 - 3\sigma_0^4}{n\sigma_0^4} tr(F_n G_n) & 0 & 0 \\ 0 & \frac{\mu_3}{2n\sigma_0^6} R(\lambda)G_n X_m \beta_m + \frac{\mu_4 - 3\sigma_0^4}{2n\sigma_0^6} tr(G_n) & 0 & \frac{\mu_4 + 3n^2\sigma_0^4 - 6n\sigma_0^4}{4n\sigma_0^8} \end{pmatrix} +$$

$$\begin{pmatrix} 0 & * & * & * \\ \frac{\mu_3}{n\sigma_0^4} R^{-1}(\lambda_0)G_n R(\lambda_0) X_m^* & \frac{\mu_3}{n\sigma_0^4}(R(\lambda_0)G_n X_m \beta_m)' R'(\lambda_0) G_n' R^{-1}(\lambda_0) & 0 & 0 \\ \frac{\mu_3}{n\sigma_0^4} F_n X_m^* & 0 & \frac{\mu_4 - 3\sigma_0^4}{n\sigma_0^4} tr(F_m^2) & * \\ \frac{\mu_3}{2n\sigma_0^6} X_m^* & 0 & \frac{\mu_4 - 3\sigma_0^4}{2n\sigma_0^6} tr(F_m) & 0 \end{pmatrix}$$

is a pairwise matrix, second, $\mu_j = E(\varepsilon_i^j), j = 2,3,4$ are the third and fourth order moments, respectively. If V_n is normally distributed, then $\Omega_{\varsigma,n} = 0$.

The second-order derivatives of the log-likelihood function with respect to ρ and λ are:

$$\frac{\partial^2 \ln L_m(\varsigma)}{\partial \rho^2} = -tr[(W_n S_n^{-1}(\rho))^2] - \frac{y_n' W_n R'(\lambda) R(\lambda) W_n y_n}{\sigma^2} = -tr(G_n^2) - \frac{y_n' W_n R'(\lambda) R(\lambda) W_n y_n}{\sigma^2},$$

$$\frac{\partial^2 \ln L_m(\varsigma)}{\partial \lambda^2} = -tr[(W_n R^{-1}(\rho))^2] - \frac{V_n R^{-1}(\lambda) W_n W_n R^{-1}(\lambda) V_n}{\sigma^2} = -tr(F_n^2) - \frac{V_n' F_n' F_n V_n}{\sigma^2}.$$

From the Lagrangian median theorem we know that

$$tr(G_n^2(\tilde{\rho}_n)) = tr(G_n^2) - 2tr(G_n^3(\bar{\rho}_n))(\tilde{\rho}_n - \rho_0),$$

$$tr(F_n^2(\tilde{\lambda}_n)) = tr(F_n^2) - 2tr(F_n^3(\tilde{\lambda}_n))(\tilde{\lambda}_n - \lambda_0).$$

From Assumption 5, we know that $G_n(\bar{\rho}_n)$ and $F_n(\bar{\lambda}_n)$ are consistently bounded in the neighborhoods of λ_0 by row sums and column sums, and satisfy

$$tr(G_n^3(\bar{\rho}_n)) = O(\frac{n}{h_n}), \quad tr(F_n^3(\bar{\lambda}_n)) = O(\frac{n}{h_n}),$$

$$y_n' W_n R(\lambda) R(\lambda) W_n y_n = O_p(\frac{n}{h_n}), \quad V_n' F_n' F_n V_n = O_p(\frac{n}{h_n}).$$

Therefore,

$$\frac{1}{n}[\frac{\partial^2 \ln L_m(\tilde{\varsigma}_n)}{\partial\rho^2} - \frac{\partial^2 \ln L_m(\varsigma_0)}{\partial\rho^2}]$$
$$= -2\frac{tr(G_n^3(\bar{\rho}))}{n}(\tilde{\rho}_n - \rho_0) + \frac{1}{n}(\frac{1}{\sigma_0^2} y_n{}' W_n R'(\lambda_0) R(\lambda_0) W_n y_n - \frac{1}{\tilde{\sigma}_n^2} y_n{}' W_n R'(\tilde{\lambda}_n) R(\tilde{\lambda}_n) W_n y_n) \quad ,$$
$$= o_p(1)$$

$$\frac{1}{n}[\frac{\partial^2 \ln L_m(\tilde{\varsigma}_n)}{\partial\lambda^2} - \frac{\partial^2 \ln L_m(\varsigma_0)}{\partial\lambda^2}]$$
$$= -2\frac{tr(F_n^3(\bar{\lambda}))}{n}(\tilde{\lambda}_n - \lambda_0) + \frac{1}{n}(\frac{1}{\sigma_0^2} V_n F_n(\tilde{\lambda}_n) F_n(\tilde{\lambda}_n) V_n - \frac{1}{\tilde{\sigma}_n^2} V_n F_n F_n V_n)$$
$$= o_p(1) \quad .$$

Then, under Lemma 2 and Lemma 3, we know that

$$\frac{1}{n}[\frac{\partial^2 \ln L_m(\tilde{\varsigma}_n)}{\partial\varsigma\partial\varsigma'} - \frac{\partial^2 \ln L_m(\varsigma_0)}{\partial\varsigma\partial\varsigma'}] \xrightarrow{P} 0,$$

and

$$\frac{1}{n}[\frac{\partial^2 \ln L_m(\varsigma_0)}{\partial\varsigma\partial\varsigma'} - E(\frac{\partial^2 \ln L_m(\varsigma_0)}{\partial\varsigma\partial\varsigma'})] \xrightarrow{P} 0.$$

Since is composed of the primary or quadratic terms, the existence of higher order moments known by Assumption 1 is known by the central limit theorem proposed by Kelejian and Prucha [34].

$$\frac{1}{\sqrt{n}}\frac{\partial \ln L_n(\varsigma_0)}{\partial\varsigma} \xrightarrow{P} N(0, \Sigma_\varsigma + \Omega_\varsigma).$$

Assumption 8 knows that Σ_ς is non-singular. The Taylor expansion for $\frac{\partial \ln L_n(\hat{\varsigma}_n)}{\partial\varsigma} = 0$ at ς_0 is:

$$\frac{\partial \ln L_n(\hat{\varsigma}_n)}{\partial\varsigma} = \frac{\partial \ln L_n(\varsigma_0)}{\partial\varsigma} + (\hat{\varsigma}_n - \varsigma_0)\frac{\partial^2 \ln L_n(\varsigma_0)}{\partial\varsigma\partial\varsigma'} = 0.$$

Then $\hat{\varsigma}_n$ can be obtained from

$$\sqrt{n}(\hat{\varsigma}_n - \varsigma_0) = -(\frac{1}{n}\frac{\partial^2 \ln L_n(\varsigma_0)}{\partial\varsigma\partial\varsigma'})^{-1}\frac{1}{\sqrt{n}}\frac{\partial \ln L_n(\varsigma_0)}{\partial\varsigma} \xrightarrow{P} -(\frac{1}{n}\frac{\partial^2 \ln L_n(\tilde{\varsigma}_n)}{\partial\varsigma\partial\varsigma'})^{-1}\frac{1}{\sqrt{n}}\frac{\partial \ln L_n(\varsigma_0)}{\partial\varsigma},$$

so

$$\sqrt{n}(\hat{\varsigma}_n - \varsigma_0) \xrightarrow{P} N(0, \Sigma_\varsigma^{-1} + \Sigma_\varsigma^{-1}\Omega_\varsigma\Sigma_\varsigma^{-1}),$$

where $\tilde{\varsigma}_n \xrightarrow{P} \varsigma_0$.

Proof of Theorem 2: According to equation (2)(3), we can obtain

$$\begin{aligned} y &= S^{-1}(\rho)[X_m\beta_m + R^{-1}(\lambda)\varepsilon] \\ &= (I_n - \rho W_n)^{-1}[X_m\beta_m + (I_n - \lambda M_n)^{-1}\varepsilon] \end{aligned}, \tag{A.8}$$

Hence, we have

$$E(Y_n) = S^{-1}(\rho)X_m\beta_m = (I_n - \rho W_n)^{-1}X_m\beta_m,$$

$$\begin{aligned} \mathrm{var}(Y_n) &= \sigma^2 S^{-1}(\rho)R^{-1}(\lambda)(R^{-1}(\lambda))'(S^{-1}(\rho))' \\ &= \sigma^2 S^{-1}(\rho)\Omega^{-1}(\lambda)(S^{-1}(\rho))' \end{aligned}.$$

By equation (6), we have

$$E(\hat{\beta}_m) = (X_m'\Omega(\lambda)X_m)X_m'\Omega(\lambda)X_m\beta_m,$$

$$\begin{aligned} \mathrm{var}(\hat{\beta}_m) &= (X_m'\Omega(\lambda)X_m)^{-1}X_m'\Omega(\lambda)S(\rho)\,\mathrm{var}(Y_n)S'(\rho)\Omega'(\lambda)X_m(X_m'\Omega(\lambda)X_m)^{-1} \\ &= \sigma_{ML}^2(X_m'\Omega(\lambda)X_m)^{-1}X_m'\Omega(\lambda)R^{-1}(\lambda)(R^{-1}(\lambda))'\Omega'(\lambda)X_m(X_m'\Omega(\lambda)X_m)^{-1} \\ &= \sigma_{ML}^2(X_m'\Omega(\lambda)X_m)^{-1}X_m'\Omega(\lambda)X_m(X_m'\Omega(\lambda)X_m)^{-1} \\ &= \sigma_{ML}^2(X_m'\Omega(\lambda)X_m)^{-1} \end{aligned}.$$

In summary, a confidence band of the slope function $\beta(t)$ of $100(1-\alpha)\%$ is

$$[\hat{\beta}(t) - \hat{\sigma}_{ML}z_{1-\frac{\alpha}{2}}\sqrt{\Phi'(t)(X_m'\Omega(\lambda)X_m)^{-1}\Phi(t)}, \hat{\beta}(t) + \hat{\sigma}_{ML}z_{1-\frac{\alpha}{2}}\sqrt{\Phi'(t)(X_m'\Omega(\lambda)X_m)^{-1}\Phi(t)}].$$

References

[34] Kelejian HH, Prucha IR. On the asymptotic distribution of the Moran I test statistic with applications. Journal of Econometrics. 2001;104(2):219-257. doi: https://doi.org/10.1016/s0304-4076(01)00064-1
